# A Long-Acting BMP-2 Release System Based on Poly(3-hydroxybutyrate) Nanoparticles Modified by Amphiphilic Phospholipid for Osteogenic Differentiation

**DOI:** 10.1155/2016/5878645

**Published:** 2016-06-09

**Authors:** Xiaochun Peng, Yunsu Chen, Yamin Li, Yiming Wang, Xianlong Zhang

**Affiliations:** Department of Orthopaedics, The Sixth Affiliated People's Hospital, Shanghai Jiaotong University, 600 Yishan Road, Shanghai 200233, China

## Abstract

We explored a novel poly(3-hydroxybutyrate) (PHB) nanoparticle loaded with hydrophilic recombinant human BMP-2 with amphiphilic phospholipid (BPC-PHB NP) for a rapid-acting and long-acting delivery system of BMP-2 for osteogenic differentiation. The BPC-PHB NPs were prepared by a solvent evaporation method and showed a spherical particle with a mean particle size of 253.4 nm, mean zeta potential of −22.42 mV, and high entrapment efficiency of 77.18%, respectively. For BPC-PHB NPs, a short initial burst release of BMP-2 from NPs in 24 h was found and it has steadily risen to reach about 80% in 20 days for* in vitro* test. BPC-PHB NPs significantly reduced the burst release of BMP-2, as compared to that of PHB NPs loading BMP-2 without PL (B-PHB NPs). BPC-PHB NPs maintained the content of BMP-2 for a long-term osteogenic differentiation. The OCT-1 cells with BPC-PHB NPs have high ALP activity in comparison with others. The gene markers for osteogenic differentiation were significantly upregulated for sample with BPC-PHB NPs, implying that BPC-PHB NPs can be used as a rapid-acting and long-acting BMP-2 delivery system for osteogenic differentiation.

## 1. Introduction

In clinical research, bone grafts originating from patients or donators have been used to repair bone defects. But it was usually confronted from the limited resource and rejection reaction in clinical practice [1, 2]. Bone tissue engineering by combination of osteogenic cell or stem cell, osteoinductive growth factor, and biomaterials is the most promising alternative to the existing therapies for bone repair and regeneration, avoiding defects of bone grafts [3].

Bone morphogenetic protein-2 (BMP-2) is one of the most important growth factors that can promote osteogenic differentiation of stem cells [4–6]. However, the clinical application of BMP-2 is limited because it is expensive and biologically unstable. No system for sustained BMP-2 delivery has been developed* in vivo* so far. Traditionally, BMP-2 gene can be transferred into cells by virus vectors [7, 8]. The potential risk of transgenic and virus infection cannot be neglected.

The use of polymer nanoparticles as a drug delivery system has been found to be useful in prolonging the duration of drugs release. Limited enhancement of drugs absorption in polymeric nanoparticles (or nanocapsules) was reported. Many nanoparticles can be produced by some biodegradable polymers, such as poly(lactic acid) (PLA), polycaprolactone (PCL), and poly(lactic-co-glycolic acid) (PLGA) [9, 10]. These polymers can easily package the hydrophobic drugs. But it is hard to achieve the high-loading of hydrophilic proteins and long-acting release for these hydrophobic polymers, due to incompatibility of polymers and proteins.

Considerable efforts have been made to improve the efficiency and reduce the quick release of BMP-2 by the combination of hydrophilic BMP-2 with hydrophobic biomaterials [11, 12]. For example, BMP-2 was blended onto PLGA by chemical technique, such as covalent binding [13, 14] or affinity binding [15, 16]. Only a tiny amount of BMP-2 can blend onto PLGA. The bioactivity of doped BMP-2 is reduced, due to the hydrogen bond breaking and structural failure during chemical process [16]. On the other hand, the double emulsion method as a moderate method has been commonly adapted to reduce the loss of BMP-2 bioactivity, but it is hard to achieve high entrapment efficiency and slow release from PLGA substrates [14, 17].

Therefore, modification of BMP-2 to disperse in polymer solution and keep its function with a moderate method is also a key of the loading and releases of bioactive BMP-2 for polymer substrates. Recently, the results showed that the amphipathic phospholipid could significantly enhance the lipophilicity of hydrophilic insulin as a drug molecule, leading to facilitating the encapsulation of hydrophilic molecules into hydrophobic nanoparticles with higher entrapment efficiency [18]. It could be a novel way to study high-loading and long-acting release of bioactive BMP-2 into polymer substrates. Qu et al. reported a novel PLGA film loaded with over 80 wt % BMP-2, which was regarded as substrate-promoting osteoblast attachment, proliferation, and differentiation for application of bone tissue engineering. Based on phospholipid as a surfactant, BMP-2 was modified as a complex (PBC) for dispersing in PLGA/dichloromethane solution [19]. It has confirmed that BMP-2 can be modified by phospholipid and reach high-loading into polymer and maintain its activity of osteoinduction.

On the other hand, poly(3-hydroxybutyrate) (PHB), which is one of the members of the microbial polyhydroxyalkanoates (PHA) family, has been studied widely in tissue engineering and treated to different forms [20–22]. Due to the strong lipophilic nature of PHB, degradation of PHB was slower than other biodegradable polymers [23]; it is an advantage for a long-acting release system. Moreover, the hydrophilic BMP-2 or other protein cannot be encapsulated perfectly into PHB nanoparticle in theory. At present, no study on application of PHB nanoparticles loaded with BMP-2 has been reported.

The aim of this study was to explore a novel PHB nanoparticle loaded with hydrophilic recombinant human BMP-2 for a long-acting delivery system for osteogenic differentiation of osteoblast OCT-1. To enhance content of BMP-2 in PHB nanoparticles, an advanced double emulsion method with amphiphilic phospholipid has been employed.

## 2. Materials and Methods

### 2.1. Materials

Poly(*β*-hydroxybutyrate) (PHB, Mw = 30 kDa) was purchased from Tianan Biologic Material Co., Ltd. (Ningbo, China). Phospholipid and poly(vinyl acetate) (PVA) were purchased from Sino pharm Co. Ltd. (Shanghai, China). Recombinant human BMP-2 was provided by Sigma Co., Ltd. (USA). Poly(vinyl acetate) (PVA, alcoholysis degree of 94.5%) was purchased from Sigma (St. Louis, USA). Other reagents were of analytical grade or better and deionized water was produced in our lab.

### 2.2. Preparation of BMP-2-Phospholipid Complex (BPC)

The BMP-2-phospholipid complex (BPC) was prepared according to a previous study [24] with some modifications. Briefly, BMP-2 and phospholipid, with a series of molar ratios of 1 : 100, were dissolved in dimethyl sulfoxide (DMSO) containing 5% (v/v) of acetic acid under magnetic stirring at 30°C in 24 h. For removing the solvent, the mixture was freezed and lyophilized for 24 h. The lyophilized BPC was sealed hermetically and stored at 4°C.

### 2.3. Preparation of Different PHB NPs

**Figure 1:**
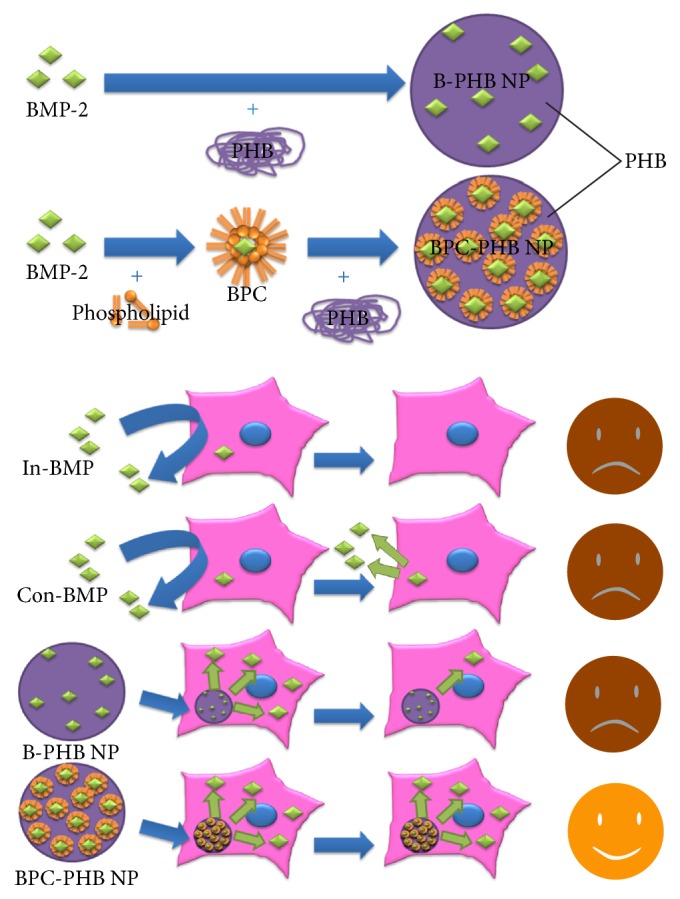
The schematic of a long-acting BMP-2 release system based on poly(3-hydroxybutyrate) nanoparticles loaded with BMP-2 modified by amphiphilic phospholipid for osteogenic differentiation. BPC: BMP-2-phospholipid complex; In-BMP: free-BMP only within initial 2 days; Con-BMP: continuous free-BMP-2 within 20 days; B-PHB NPs: PHB nanoparticles loaded with pure BMP-2; B-PHB NPs: PHB nanoparticles loaded with pure BMP-2; BPC-PHB NPs: PHB nanoparticles loaded with BPC.

The BPC-loading PHB nanoparticles (BPC-PHB NPs) were produced by an emulsion-solvent evaporation method, according to the schematic in Figure 1. Briefly, 12.5 g of BPC and 250 mg of PHB were simultaneously dissolved in 5 mL dichloromethane for an organic phase. As aqueous phase, poly(vinyl acetate) (PVA) of 1 g was dissolved in 100 mL distilled water at 80°C. Above aqueous phase was subsequently mixed into the organic phase, followed by sonication for 10 times (working for 5 s and resting for 5 s per time) at a power of 800 W in ice water bath. The emulsion was evaporated under rotary evaporator at 30°C for 30 min to remove the organic solvent and centrifuged to wash residuary PVA and F68. The BMP-2-loading PHB nanoparticles without phospholipids (B-PHBNPs) and pure PHB nanoparticles without BMP-2 (PHBNPs) were included as two controls. They were produced by similar emulsion-solvent evaporation method, with pure BMP-2 and nothing, respectively, for replacement of BPC mixing into dichloromethane as organic phase.

### 2.4. Characters of Different PHB NPs

BPC-PHB NPs and B-PHB NPs were observed by scanning electron microscopy (SEM), respectively. The suspension of NPs was placed on a glass surface and air-drying. The glass was coated with gold in an auto fine coater (JFC-1600, JEOL, Japan) for 10 min at 10 mA, finally, followed by examination under a SEM (Hitachi, S-4800, Japan). With dynamic light scattering (DLS) and electrophoretic light scattering (ELS) technology, the mean particle size, size distribution, and zeta potential of the BPC-PHB NPs and B-PHB NP were characterized by a Zetasizer Nano ZS90 instrument (Malvern Instruments Ltd., UK), using water as a dispersant at 25°C with each cycle of the measurement automatically determined by the instrument system. The particle size was displayed by intensity distribution, and the size distribution was expressed by polydispersity index (PDI).

### 2.5. Entrapment Efficiency (EE) Studies

A modified centrifugation method was to evaluate the entrapment efficiency of BPC-PHB NPs and B-PHB NP [25]. The free-BMP-2 (*W*_*f*_), which was not packaged into NPs and dispersed in supernatant, was analyzed by an ELISA Kit. In above preparation of BPC-PHB or B-PHB NPs, another equal BPC was dissolved in the same volume PVA of 0.1% (w/v) and then treated by the same method described by Section 2.4. The content of BMP-2 was measured by the same ELISA Kit and defined to total amount of BMP-2 (*W*_*t*_). Therefore, the entrapment efficiency could be calculated through the following equation:

(1) $$\begin{array}{c} \text{EE }\left(\;\%\;\right)=\frac{\left(W_{t}-W_{f}\right)}{W_{t}}\times 100. \end{array}$$

### 2.6. *In Vitro* Release Studies of BPC-PHB NPs

The* in vitro* release of the BMP-2 from nanoparticle was studied using the dialysis method. The dialysis bags with a molecular weight cut-off of 50 kDa were used to retain BPC-PHB NPs in the bags, allowing the released BMP-2 to permeate into the release medium (PBS, pH = 7.2, Gibco, Thermo Fisher, USA); 1 mL of BPC-PHB NPs was added in a dialysis bag and tightly bundling the end. It was immersed in 8 mL of release medium and shaken in a shaker at 50 rpm at 37°C. At fixed time intervals, the release medium containing free-BMP-2 was collected and replaced with 8 mL fresh medium. The collected sample was diluted properly and centrifuged at 12000 rpm for 10 min. The content of released BMP-2 in supernatant was determined by BMP-2 ELISA Kit. B-PHB NPs as a control was also analyzed by same way for release studies* in vitro*.

### 2.7. Cell Culture and Biocompatibility

To study the biocompatibility of BPC-PHB NPs, the human osteoblast-like cell, OCT-1, was employed. The OCT-1 cell is an osteoblast-like cell which is derived from osteocalcin promoter-driven SV-40 T-antigen transgenic mouse calvarias and supplied by Chinese Academy of Sciences (Shanghai, China). OCT-1 cells were incubated at 37°C in DMEM supplemented with 10% fetal bovine serum (FBS), 100 U/mL penicillin, and 80 *μ*g/mL streptomycin in a 5.0% carbon dioxide incubator.

To determine the biocompatibility, OCT-1 cells with BPC-PHB NPs were cocultivated for 5, 10, 15, and 20 days, respectively and then detected with a CCK-8 Kit. Briefly, 2 mL fresh DMEM medium containing 0.2 mL CCK-8 solutions was added to each sample and continually incubated at 37°C for 2 h. All microspheres must be immersed in DMEM medium. The absorbance was measured at 450 nm by microplate reader (Multiskan MK3, Thermo Lab systems, Finland). For each sample, six parallel replicates were read in this study.

### 2.8. Alkaline Phosphatase Assay

To explore alkaline phosphatase (ALP) expression in OCT-1 cells with BPC-PHB NPs for long-term release of BMP-2, the cells were cocultivated continuously for 5, 10, 15, and 20 days and the medium was changed every day. For osteogenesis difference detection resulting from long-term release and of initial burst release of BMP-2, another two samples were employed, which were treated by continuous BMP-2 within 20 days and only within initial 2 days and defined as Con-BMP and In-BMP. For simulation of cell metabolism and* in vivo* blood-supply, DMEM was replaced by fresh DMEM every day in the whole process.

The cells from all samples were then washed by PBS twice and suspended in 0.1% Triton X-100 for 10 min. After OCT-1 cells lysis, the solution was centrifuged and the supernatants were collected for measuring the ALP activity. ALP activity was determined with ALP Activity Kit (Jiangcheng, Nanjing, China), using disodium phenyl phosphate as the substrate. The absorbance at 520 nm was measured with the microplate reader. And total protein content was determined at 595 nm, using a Protein Assay Kit (Tiangen, Beijing, China). The cells with B-PHB NPs and cells with nothing as controls were treated by same method.

### 2.9. Osteogenic gene Markers Analysis

To investigate differentiation and matrix mineralization of OCT-1 cells with BPC-PHB NPs or B-PHB NPs for cultivating at day 20, the expression levels of osteogenic gene markers were estimated by quantitative PCR analysis, referred to in previous study [26]. Using the QuantiTect*™* SYBR Green PCR Kit (Qiagen, Hilden, Germany), we focused on 8 kinds of gene markers, collagen type-1 (COL-1), matrix Gla-protein (MGP), Runt-related transcription factor 2 (Runx2), osteocalcin (OCN), osteopontin (OPN), and osteoprotegerin (OPG), in this study, respectively. And the primers used were shown in Table 1. According to the instruction of Kit, 1 mg of the total RNA of cells for each sample was purified to perform synthesis of complementary DNA (cDNA). Similarly, the DMEM was replaced by the fresh DMEM every day in this test and the PHB NPs, Con-BMP, and In-BMP acted as controls.

### 2.10. Immunohistochemical Analysis

For immunohistochemical analysis, the OCT-1 cells treated by all samples on day 20 were fixed by 4% paraformaldehyde and immunostained using anti-human osteocalcin antibody for rabbit IgG (Molecular Probes, Thermo Fisher, USA) as described previously [27]. The secondary antibody was Alexa Fluor 488-conjugated anti-rabbit IgG (Molecular Probes, Thermo Fisher, USA). The negative control was treated with the isotype IgGs to replace primary antibodies. The nucleus was fixed by 4% paraformaldehyde and stained by DAPI solution (Molecular Probes, Thermo Fisher, USA). And the colored OCT-1 was then observed by confocal laser scanning microscope (CLSM, TCS SP5, Leica, Germany) for exciting light at 488 nm and 405 nm.

### 2.11. Statistical Analysis

All data were presented as the mean value plus standard deviation (mean ± SD). Statistical comparisons were performed by Student's *t*-test and performed by Graph-Pad Prism 5 (Graph-Pad, La Jolla, CA), with a confidence level of 95% (*p* < 0.05) considered statistically significance.

## 3. Results

### 3.1. Characterization of BPC-PHB NPs

**Figure 2:**
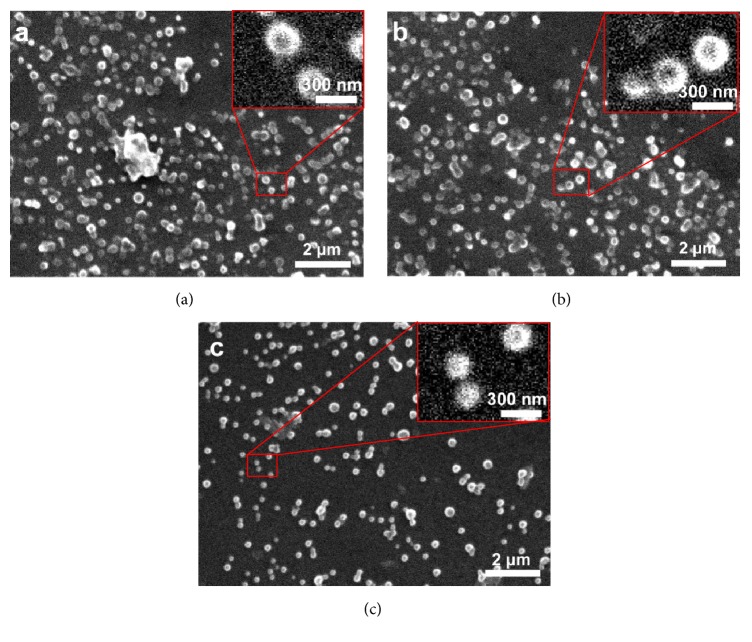
The morphology of (a) B-PHB NPs, (b) BPC-PHB NPs, and (c) PHB NPs. B-PHB NPs: the PHB nanoparticles loaded with pure BMP-2 without phospholipid; BPC-PHB NPs: the PHB nanoparticles loaded with BPC-PHB; PHB NPs: the PHB nanoparticles.

BPC-PHB NPs containing BPCs and B-PHB NPs without phospholipid were observed by SEM, respectively. They both showed mostly spherical nanoparticle and were well distributed in size (Figure 2). Attentively, some conjugations as big chunk can be observed in B-PHB NPs (Figure 2(a)); however, similar conjugations were found in PHB NPs and BPC-PHB NPs (Figures 2(b) and 2(c)). It explained that pure BMP-2 could affect the dispersibility and uniformity of PHB NPs, on account of the hydrophilia. And adding of phospholipids could improve that. The characterizations of BPC-PHB NPs containing different BPCs and B-PHB NP loading unmodified BMP-2, which were the mean particle size, polydispersity index (PDI), zeta potential, and entrapment efficiency (EE), were characterized in Table 2, respectively.

Among three types of BPC-PHB NPs and B-PHB NP, their size and PDI fluctuated slightly but zeta potential was significantly raised with increase of phospholipid composition in BPCs. In addition, the entrapment efficiency of BPC-PHB NPs was 77.18%, also significantly more than that of BPC-PHB NPs of 12.53%.

### 3.2. *In Vitro* Release Studies of BPC-PHB NPs

**Figure 3:**
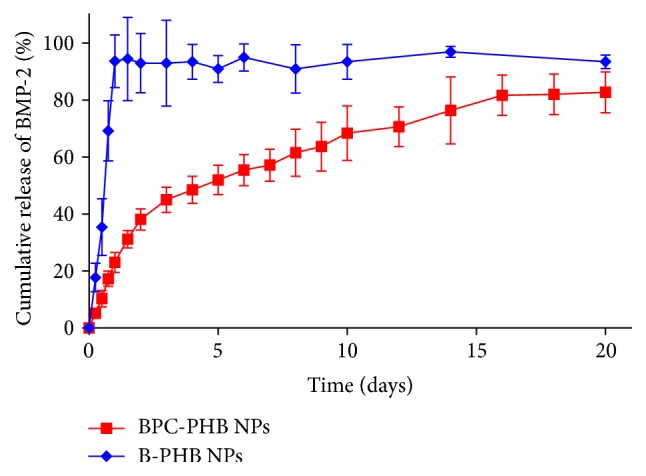
The* in vitro* release profile of BMP-2 from BPC-PHB NPs and B-PHB NPs in PBS at 50 rpm at 37°C (*n* = 4). B-PHB NPs: the PHB nanoparticles loaded with pure BMP-2 without phospholipid; BPC-PHB NPs: the PHB nanoparticles loaded with BPC-PHB.

The* in vitro* release of BMP-2 from BPC-PHB NPs and B-PHB NPs was studied in PBS up to 20 days, using the dynamic dialysis method (Figure 3). BPC-PHB NPs showed a long-acting and sustained BMP-2 release. Only about 10% of BMP-2 was released in the initial 24 h, followed by a sustained and slow release, as evidenced by about 80% of BMP-2 being released on 20th day. In contrast, B-PHB NPs showed a burst release of BMP-2 and more than 90% of loaded BMP-2 was released within 24 h. This is a conventional phenomenon for NPs loaded with proteins. However, BPC-PHB NPs did not have similar difference on day 1 (Figure 3). The BMP-2 release from BPC-PHB NPs was mostly slow-growth, except burst release of initial 24 h. In other words, the release profile of B-PHB NPs can be divided into two typical phases: burst release phase of 24 h and sustained release phase after 24. And approximately 80% of BMP-2 was cumulatively released from BPC-PHB NPs in 20 days.

### 3.3. Biocompatibility

**Figure 4:**
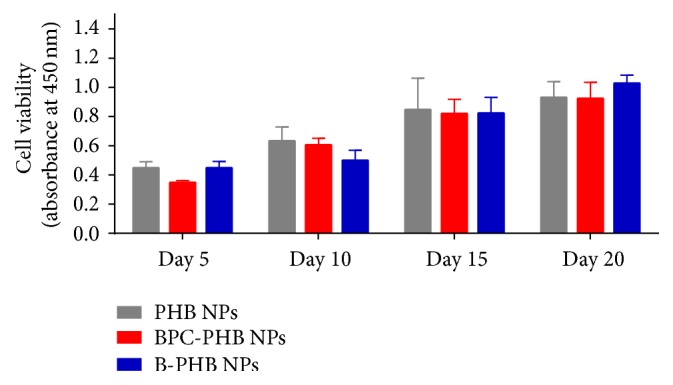
Cell viability of OCT-1 cells treated by various cultures within samples within 20 days, respectively (*n* = 6). B-PHB NPs: the PHB nanoparticles loaded with pure BMP-2 without phospholipid; BPC-PHB NPs: the PHB nanoparticles loaded with BPC-PHB; PHB NPs: the PHB nanoparticles.

After cells cultivation with BPC-PHB NPs, B-PHB NPs, and PHB NPs in 1 day, the original DMEM was replaced by fresh DMEM to remove nonuptake NPs. And the cells were cultivated within 20 days. The cell viability of all samples was estimated by CCK-8 Kit, respectively (Figure 4). No obvious difference was observed of these samples (i.e., *p* > 0.05 in statistics) as compared to the cells without NPs, sample TCP. It illustrated that the interference of various NPs into cells was negligible. In other words, the BPC-PHB NPs, B-PHB NPs, and PHB NPs all have good biocompatibility.

### 3.4. Differentiation of OCT-1 Cells on BPC-PMSs

#### 3.4.1. ALP

**Figure 5:**
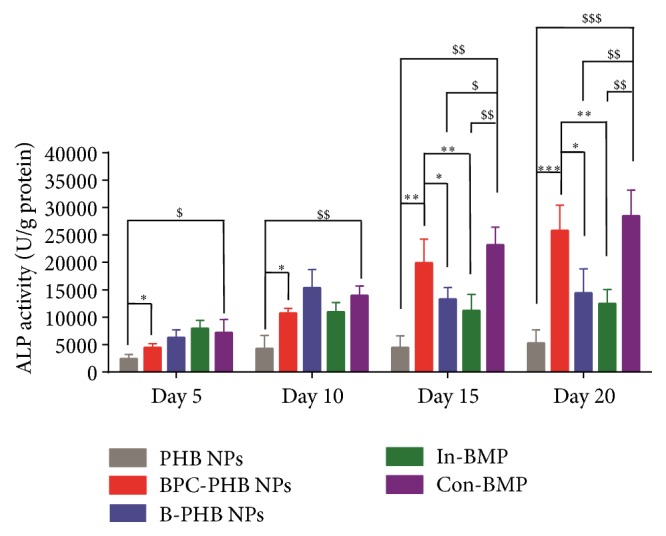
The ALP activity of OCT-1 cells treated by various cultures within samples within 20 days, respectively. Statistically significant difference against the ALP activity of BPC-PHB NPs, ^*∗*^ *p* < 0.05, ^*∗∗*^ *p* < 0.01, and ^*∗∗∗*^ *p* < 0.005; statistically significant difference against the ALP activity of Con-BMP, ^$^ *p* < 0.05, ^$$^ *p* < 0.01, and ^$$$^ *p* < 0.005. (*n* = 6). B-PHB NPs: the PHB nanoparticles loaded with pure BMP-2 without phospholipid; BPC-PHB NPs: the PHB nanoparticles loaded with BPC-PHB; PHB NPs: the PHB nanoparticles. In-BMP: free-BMP only within initial 2 days; Con-BMP: continuous free-BMP-2 within 20 days.

As shown in Figure 5, the activity of ALP in OCT-1 cells increased significantly for all cells samples with different treatments within 20 days. Apparently, for four samples treated by BMP-2, that is, BPC-PHB NPs, B-PHB NP, In-BMP, and Con-BMP, respectively, ALP activities increased fast and higher than B-PHB NPs of 2501 U/g protein (*p* < 0.05). After 10 days in culture, ALP activity gap of BPC-PHB NPs and B-PHB NPs started to enlarge, and final ALP activity of them was 12537 U/g protein and 5327 U/g protein, respectively.

On the other hand, the two controls of BMP-2 solution, samples of continuous BMP-2 (Con-BMP) and Initial BMP-2 (In-BMP), showed similar tendency to BPC-PHB NPs and B-PHB NPs. Due to the continuous BMP-2 supply, the ALP activity of BPC-PHB NPs and continuous BMP-2 increased steadily within 20 days. On the contrary, the samples of B-PHB NPs and initial BMP-2, only showed an initial ALP activity increase and paused within the middle and later periods of differentiation. It could be a reason that initial BMP-2 could not maintain a long-acting osteogenic differentiation.

#### 3.4.2. Quantitative PCR

**Figure 6:**
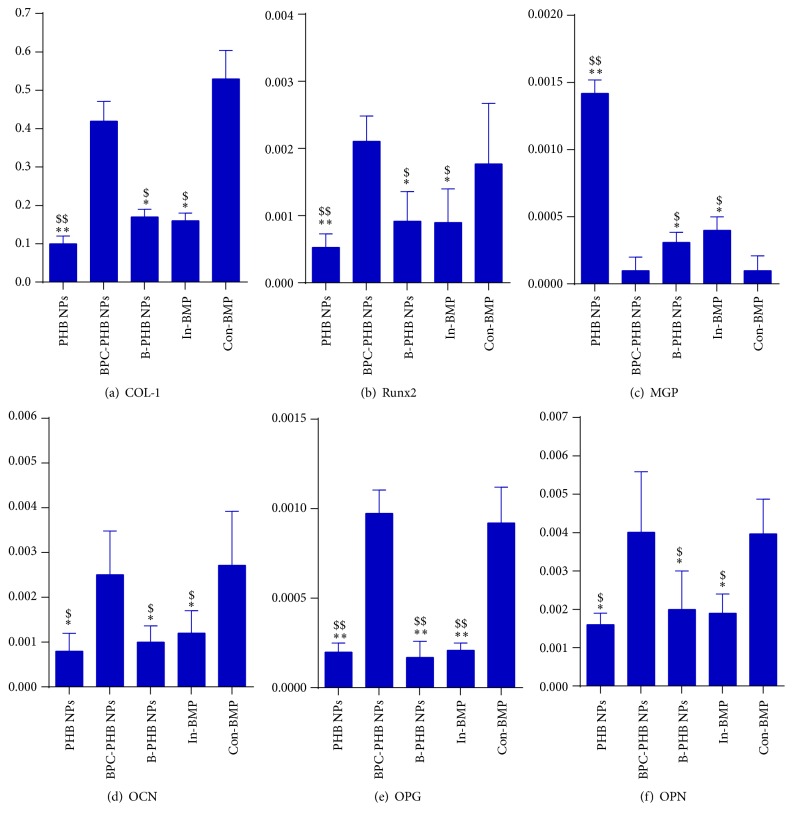
Quantitative PCR analysis of osteogenic gene markers expression by OCT-1 cells treated by various samples on day 20. The *y*-axis represents the relative expression (2^−ΔCT^) normalized to the expression level of the house keeping gene *β*-Actin. (a) COL-1: collagen type-1; (b) Runx2: runt-related transcription factor; (c) MGP: matrix Gla-protein; (d) OCN: osteocalcin; (e) OPG: osteoprotegerin; (f) OPN: osteopontin (*n* = 6). B-PHB NPs: the PHB nanoparticles loaded with pure BMP-2 without phospholipid; BPC-PHB NPs: the PHB nanoparticles loaded with BPC-PHB; PHB NPs: the PHB nanoparticles. In-BMP: free-BMP only within initial 2 days; Con-BMP: continuous free-BMP-2 within 20 days. Statistically significant difference against BPC-PHB-NPs, ^*∗*^ *p* < 0.05, ^*∗∗*^ *p* < 0.01; statistically significant difference against Con-BMP, ^$^ *p* < 0.05, ^$$^ *p* < 0.01. (*n* = 6).

In the meantime, 6 types of biomarkers for osteogenesis, which contained collagen type-1 (COL-1), matrix Gla-protein (MGP), Runt-related transcription factor 2 (Runx2), osteocalcin (OCN), osteopontin (OPN), and osteoprotegerin (OPG), were assessed by SYBR green PCR and shown in Figure 6, respectively. On day 20 of OCT-1 cell cultivation, expression of COL-1 in cells treated by BPC-PHB NPs was upregulated significantly with increase of BMP-2 from NPs, which was similar to sample treated by continuous BMP-2.

Different from upregulated COL-1, MGP, a calcification inhibitor, showed the remarkable downregulation tendency (*p* < 0.01) by cells with BPC-PHB NPs in comparison with B-PHB NPs and PHB NPs. Runx2, a multifunctional transcription factor that controls skeletal development, was also significantly higher than that of the negative control (B-PHB NPs and PHB NPs) (Figure 6). Moreover, OCN and OPN were both significantly upregulated (*p* < 0.01) by cells treated by BPC-PHB NPs and were closed to the samples of Con-BMP-2, due to the continuous BMP-2 supply.

Surprisingly, OPG, an osteoclastogenesis inhibitory factor, also was upregulated significantly for sample BPC-PHB NPs. In Figure 6, it was not difficult to find that contents of 6 markers on BPC-PHB NPs and Con-BMP were similar. It supported the result that there was no difference for the BMP-2 supplies between them. Simultaneously, it implied that the phospholipid as a biosurfactant did not disturb the differentiation of OCT-1 cells from BPC-PHB NPs.

#### 3.4.3. Immunohistochemical Analysis of OCN

**Figure 7:**
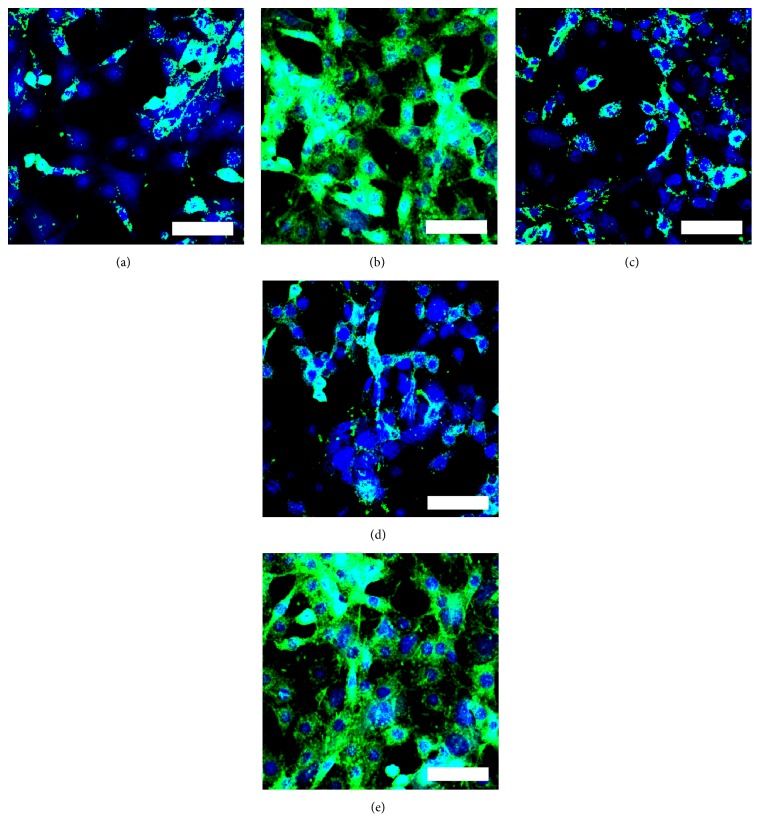
Immunohistochemical analysis of OCN, the OCT-1 cells on day 20 with (a) PHB NPs, (b) BPC-PHB NPs, (c) B-PHB NPs, (d) In-BMP, and (e) Con-BMP, respectively. B-PHB NPs: the PHB nanoparticles loaded with pure BMP-2 without phospholipid; BPC-PHB NPs: the PHB nanoparticles loaded with BPC-PHB; PHB NPs: the PHB nanoparticles. In-BMP: free-BMP only within initial 2 days; Con-BMP: continuous free-BMP-2 within 20 days. The bar is 40 *μ*m.

For immunohistochemical analysis of OCN, the OCT-1 cells treated by all samples on day 20 were stained and observed by CLSM. As shown in Figure 7, the OCT-1 cells with BPC-PHB NPs had more expression of OCN and were similar to sample of Con-BMP. The others showed remarkably lower OCN than BPC-PHB NPs under the same conditions. It further illustrated that osteogenesis differentiation effect of BPC-PHB NPs is closed to continuous BMP-2 supplement.

## 4. Discussion

In previous studies, most proteins (e.g., insulin) or growth factors (e.g., BMP-2) were added directly into polymers by the double emulsion method; however, low entrapment efficiency and the loss of these proteins during fabrication process could not be avoided [10, 28–30]. Certainly, some surfactants (e.g., Tween 20) and stabilizer (e.g., polyvinyl alcohol) attempted to improve the above problems in double emulsion method [31–34].

Since 2008, phospholipid, a natural amphipathic molecule constituted cytomembrane, was shown to enhance the lipophilicity of protein by formation of phospholipid mixture [18, 25, 35]. It is a prerequisite and challenge to entrap hydrophilic proteins or growth factors directly dispersed in polymers, due to the strong lipophilicity of this formation of phospholipid mixture. Subsequently, this technique was utilized to produce the rapid-acting, long-acting insulin formulation based on phospholipid complex loaded PHBHHx nanoparticles (INS-PLC-NPs) for diabetes treatment [18].

Inspired by above reports, we prepared a novel PHB nanoparticle loaded with recombinant human BMP-2-phospholipid complex (BPC) to achieve the rapid-acting and long-acting BMP-2 formulation. It was a challenge to entrap hydrophilic BMP-2 directly into PHB nanoparticles which is a strong lipophilicity biomaterial, similarly to PHBHHx. Using an emulsion-solvent evaporation method, the novel PHB nanoparticles with the high encapsulation efficiency of BMP-2 were successfully prepared, due to the phospholipid modification (Table 2). It has a distinct difference among three NPs, BPC-PHB NPs, and two controls: pure PHB NPs and B-PHB NPs with pure BMP-2. They have similar particle sizes and zeta potential. However, the phospholipids enhanced the encapsulation of BMP-2 into PHB matrix, which is remarkable. For PHBHHx and PLGA, phospholipids had same acceleration to increase content of proteins in polymers [18, 25, 35].

In addition to entrapment efficiency, the sustaining* in vitro* release profile of BMP-2 also was an important factor for BMP-2 loading PHB NPs. Most studies illustrated the significantly faster* in vitro* release rate including the initial burst release of BMP-2 from particles or microspheres [14, 30, 36–39]. The relatively fast release of BMP-2 could be attributed to a large amount of BMP-2 adsorbed on the surface of particles rather than inside. Except particles loaded with BMP-2, another form of polymer scaffolds, showed similar results in previous studies. Nie et al. proved the percentage of BMP-2 released slightly less than 25% after more than 360 h (or 15 days) from 3D fibrous PLGA/HAp mixture scaffold for BMP-2 delivery* in vitro* release study [40]. Kempen et al. compared four scaffolds including PLGA loading BMP-2, indicating that about 30% BMP-2 accumulated in 2-3 days [41, 42]. To sum up, no reports have described PHB or other PHAs packaged with abundant BMP-2 for long-acting release of BMP-2. In this study, the approximately 80% of BMP-2 was released from BPC-PHB NPs in 20 days, and the released BMP-2 showed slowly increase tendency (Figure 3).

As shown in Figure 4, the similar results of the cell growth of OCT-1 with different NPs, explained that BPC-PHB NPs are of good biocompatibility and the interference of various NPs into cells was negligible. However, the osteogenic differentiation advantage of BPC-PHB NPs is obvious, because BMP-2 promoted osteoblast growth and proliferation was widely accepted. As the most typical bioactive marker on osteogenesis, the expression of alkaline phosphatase (ALP) in osteoblasts must be evaluated* in vitro* or* in vivo*. As we expected, ALP analysis showed more benefits of PBM-PMSs to improve OCT-1 differentiation. This may be also due to the abundant BMP-2 in BPC-PHB NPs and their sustaining BMP-2 release.

Due to the continuous BMP-2 supply, the ALP activity of BPC-PHB NPs and continuous BMP-2 (Con-BMP) increased steadily within 20 days. On the contrary, the samples of B-PHB NPs and initial BMP-2 (In-BMP) only showed an initial ALP activity increase and paused within the middle and later periods of differentiation. It could be a reason that initial BMP-2 could not maintain a long-acting osteogenic differentiation as shown in Figure 1.

In addition, based on the gene markers analysis data, osteogenic differentiation of OCT-1 was confirmed on BPC-PHB NPs. Among these gene markers, COL-1, MGP, OCN, Runx2, and OPN represented the fact that osteogenic differentiation was significantly upregulated due to the sustained stimulation of BMP-2 in previous studies [43]. Therefore, the ideal scaffolds with BMP-2 release should show same upregulation status for these markers. Inversely, the calcification inhibitor, MGP, should be downregulated, because of the BMP-2 existing in OCT-1 cells. The levels of up- or down-regulation of biomarkers were controlled by BMP-2. In other words, more BMP-2 could result in higher-level regulation than low BMP-2. As expected, COL-1, MGP, OCN, and OPN of sample BPC-PHB NPs are higher and MGP of that sample is lower on day 20. In brief, it was not difficult to find that contents of 6 markers on BPC-PHB NPs and Con-BMP were similar. It supported the result that there was no difference for the BMP-2 supplies between them. Hence, it implied that the phospholipid as a biosurfactant did not disturb the differentiation of OCT-1 cells from BPC-PHB NPs. The results of immunohistochemical analysis of OCN also support that osteogenesis differentiation effect of BPC-PHB NPs is closed to continuous BMP-2 supplement.

## 5. Conclusions

By modified solvent evaporation method with BMP-2 phospholipid complex, biodegradable PHB nanoparticles loaded with abundant BMP-2 with phospholipid (BPC-PHB NPs) were successfully produced in this study. The BPC-PHB NP had small particle size and high BMP-2 entrapment efficiency. For* in vitro* release test, BPC-PHB NPs showed typical slow release curve which had a short initial burst release that passed 24 h and steadily rose to reach about 80% of BMP-2 release rate in 20 days. It avoided serious burst release of BMP-2 such as PHB NPs directly loading BMP-2 without PL (B-PHB NPs), so that it maintained the content of BMP-2 for a long-term osteogenic differentiation. With BPC-PHB NPs, the OCT-1 cells have high ALP activity and some gene markers for osteogenic differentiation were significantly upregulated.

## Figures and Tables

**Table 1:** Primers used for SYBR green polymerase chain reaction (RT-PCR) in this study.

Gene markers^a^	Primer sequences	
	Forward	Reverse
COL-1	5′-GACGAAGACATCCCACCAAT-3′	5′-AGATCACGTCATCGCACAAC-3′
Runx2	5′-CCCAGATCATGTTTGAGACCT-3′	5′-CCTCGTAGATGGGCACAGT-3′
MGP	5′-CAAGAGAGGATCCGAGAACG-3′	5′-CGCTTCCTGAAGTAGCGATT-3′
OCN	5′-GTGCAGCCTTTGTGTCCAA-3′	5′-GCTCACACACCTCCCTCCT-3′
OPN	5′-ACTGATTTTCCCACGGACCT-3′	5′-TCAGGGTACTGGATGTCAGG-3′
OPG	5′-GGGGACCACAATGAACAACT-3′	5′-AGCTGATGAGAGGTTTCTTCG-3′

^a^COL-1: collagen type-1; Runx2: runt-related transcription factor; MGP: matrix Gla-protein; OCN: osteocalcin; OPG: osteoprotegerin; OPN: osteopontin.

**Table 2:** Characterization of BPC-PHB NPs prepared using different BPCs and B-PHB NPs prepared using pure BMP-2 without phospholipids, respectively (*n* = 6).

Samples	Size (nm)	PDI	Zeta potential (mV)	EE (%)
PHB NPs	242.1 ± 5.2	0.125 ± 0.012	−25.21 ± 2.19	—
B-PHB NPs	251.4 ± 8.1	0.155 ± 0.015	−12.52 ± 1.54	12.53 ± 1.62
BPC-PHB NPs	253.4 ± 7.1	0.143 ± 0.018	−22.42 ± 2.14	77.18 ± 6.39
